# Transcriptome analysis of paired primary colorectal carcinoma and liver metastases reveals fusion transcripts and similar gene expression profiles in primary carcinoma and liver metastases

**DOI:** 10.1186/s12885-016-2596-3

**Published:** 2016-07-26

**Authors:** Ja-Rang Lee, Chae Hwa Kwon, Yuri Choi, Hye Ji Park, Hyun Sung Kim, Hong-Jae Jo, Nahmgun Oh, Do Youn Park

**Affiliations:** 1 Department of Pathology Pusan National University Hospital, Pusan National University School of Medicine, Seo-Gu Busan, 602-739 Korea; 2 BioMedical Research Institute Pusan National University Hospital, Seo-Gu Busan, Korea; 3 Department of Surgery Pusan National University Hospital, Pusan National University School of Medicine, Seo-Gu Busan, Korea

**Keywords:** Colorectal cancer, RNA-seq, Expression profiling, Gene fusion

## Abstract

**Background:**

Despite the clinical significance of liver metastases, the difference between molecular and cellular changes in primary colorectal cancers (CRC) and matched liver metastases is poorly understood.

**Methods:**

In order to compare gene expression patterns and identify fusion genes in these two types of tumors, we performed high-throughput transcriptome sequencing of five sets of quadruple-matched tissues (primary CRC, liver metastases, normal colon, and liver).

**Results:**

The gene expression patterns in normal colon and liver were successfully distinguished from those in CRCs; however, RNA sequencing revealed that the gene expression between primary CRCs and their matched liver metastases is highly similar. We identified 1895 genes that were differentially expressed in the primary carcinoma and liver metastases, than that in the normal colon tissues. A major proportion of the transcripts, identified by gene expression profiling as significantly enriched in the primary carcinoma and metastases, belonged to gene ontology categories involved in the cell cycle, mitosis, and cell division. Furthermore, we identified gene fusion events in primary carcinoma and metastases, and the fusion transcripts were experimentally confirmed. Among these, a chimeric transcript resulting from the fusion of *RNF43* and *SUPT4H1* was found to occur frequently in primary colorectal carcinoma. In addition, knockdown of the expression of this *RNF43-SUPT4H1* chimeric transcript was found to have a growth-inhibitory effect in colorectal cancer cells.

**Conclusions:**

The present study reports a high concordance of gene expression in the primary carcinoma and liver metastases, and reveals potential new targets, such as fusion genes, against primary and metastatic colorectal carcinoma.

**Electronic supplementary material:**

The online version of this article (doi:10.1186/s12885-016-2596-3) contains supplementary material, which is available to authorized users.

## Background

Colorectal cancer is a commonly occurring cancer worldwide [1]. Metastatic colorectal cancer is clinically significant, as colorectal cancer is one of the major causes of cancer-related deaths [2]. Metastatic progression in colorectal cancer is a multistep process, beginning with the formation of adenomatous polyps, which develop into locally invasive tumors [3]. This process involves phenotypic changes associated with the acquisition of new functions, such as cell-type transition, cell migration, and tissue invasion in the tumor cells [3]. An improved understanding of the molecular alterations associated with metastatic progression may contribute to the development of novel and effective targeted therapies for colorectal cancer [4].

Gene expression profiling provides a scalable molecular method for investigating genetic variation, associated with ectopic gene expression, in tumors. Also, the identification of differentially expressed genes offers great potential for the discovery of clinically useful biomarkers in tumor cells. The complexity of the cancer transcriptome is attributable to differential pre-mRNA processing, including alternative promoter and splicing, which is involved in the production of cancer-specific transcripts and proteins [5]. Fusion transcripts are common cancer-specific RNAs, which are obtained by genomic rearrangements or transcription-mediated mechanisms, such as novel *cis* or *trans* splicing [6]. The formation of gene fusions may lead to the disruption of tumor suppressor genes or the activation of oncogenes, thereby triggering tumorigenesis [7]. Furthermore, fusion transcripts and proteins have been useful in cancer diagnosis, prognosis, and direct target therapy.

Massively parallel RNA sequencing (RNA-seq) is a useful method for annotation of the cancer transcriptome with great efficiency and high resolution [8] RNA-seq has enabled a comprehensive understanding of the complexity of the cancer transcriptome, via genome-wide expression profiling and identification of novel and fusion transcripts [9]. Recently, RNA-seq has been used to annotate the cancer transcriptome in breast [10], lung [11], gastric [12], and colorectal cancers [13, 14]. However, despite the availability of high-throughput sequencing technology, the transcriptional differences including fusion genes between primary colorectal carcinomas and liver metastases not fully understood.

In this study, we compared the transcriptomes of five sets of quadruple-matched tissues (primary carcinomas, liver metastases, normal colon, and liver). First, we found a similar gene expression pattern between primary and metastatic colorectal carcinoma. Second, we identified a novel gene fusion event specifically in primary and metastatic colorectal cancer tissue, and experimentally confirmed the fusion product. In addition, we demonstrated the cell growth-promoting effect of this fusion transcript.

## Methods

### Collection of specimens

Matched fresh-frozen samples, including 5 paired primary, metastatic colorectal carcinoma, normal colon and liver, who received resection of the primary tumor at the Korean National Biobank of Pusan National University Hospital (PNUH) were obtained from the Korean National Biobank of PNUH. This series of studies was reviewed and approved by Institutional Ethics Committees of Pusan National University Hospital. All of the patients that were used in this study and their characteristics were summarized in Additional file 1: Table S1.

### cDNA library preparation and high-throughput paired-end RNA sequencing

Total RNA was isolated from fresh-frozen tissues of the conditioned volunteers and patients (NC, normal colon; PC, primary colon carcinoma; LM, colon-liver metastases; NL, normal liver) using TRIzol reagents (Invitrogen, USA), and subsequently treated with RNase-free DNaseI for 30 min at 37 °C, to remove residual DNA. Libraries were prepared according to the standard Illumina mRNA library preparation (Illumina Inc, USA). Briefly, Purified mRNA was fragmented in fragmentation buffer and we obtained short fragments of mRNA. These short fragments served as templates to synthesize the first-strand cDNA, using random hexamer primers. The second-strand cDNA was synthesized using buffer, dNTPs, RNase H, and DNA polymerase I, respectively. Double-stranded cDNAs were purified with QiaQuick PCR extraction kit (Qiagen Inc, USA) and resolved with EB buffer. Following the synthesis of 2nd strand, end repair, and addition of a single A base, Illumina sequencing adaptors were ligated onto the short fragments.

The concentration of each library was measured by real-time PCR. Agilent 2100 Bioanalyzer was used to estimate insert size distribution. Constructed libraries were sequenced (90 cycles) using Illumina HiSeq^TM^ 2000 (Illumina Inc), according to the manufacturer’s instructions. HiSeq Control Software (HCS v1.1.37) with RTA (v1.7.45) was used for management and execution of the HiSeq^TM^ 2000 runs.

### RNA-seq data processing

Images generated by HiSeqTM2000 were converted into nucleotide sequences by a base calling pipeline and stored in FASTQ format, and the dirty raw reads were removed prior to analyzing the data. Three criteria were used to filter out dirty raw reads: Remove reads with sequence adaptors; Remove reads with more than 5 % ‘N’ bases; Remove low-quality reads, which have more than 50 % QA ≤ 10 bases. All subsequent analyses were based on clean reads.

Clean reads were mapped to reference *Homo sapiens* transcriptome sequences from the UCSC website (hg19), using Bowtie2 and Tophat 2.0.1. Mismatches of no more than 3 bases were allowed in the alignment for each read. Reads matched with reference rRNA sequences were also mapped and removed. To annotate gene expression, fragments per kb per million fragments (FPKM) values of each gene were calculated, and differentially expressed genes (DEGs) were extracted using this value. The formula for calculating FPKM value was defined as below:

$$ \mathrm{FPKM}=\frac{10^9C}{NL/{10}^3} $$

In this formula, C represents the number of reads uniquely mapped to the given gene, N is the number of reads uniquely mapped to all genes, and L is the total length of exons from the given gene. For genes with more than one alternative transcript, the longest transcript was selected to calculate the FPKM value.

### Expression profiling and analysis of differential gene expression

For clustering, genes with median of RPKM < 1.0 and coefficient of variation (CV) < 0.7 were excluded to remove genes non-informative. This resulted in a total of 7744 unique genes. Log_2_ transformation and additional normalization was applied. Then, hierarchical clustering was done by Gene Cluster 3.0 with default parameters, correlation (uncentered), and complete linkage [15]. The differential expression *P*-values were adjusted using the false discovery rate (FDR) by the Benjamini and Hochberg procedure and set a cutoff of FDR < 0.05. Analyzed genes were functionally annotated in accordance with the Gene Ontology (GO) using the DAVID bioinformatics tool (http://david.abcc.ncifcrf.gov) [16].

### Candidate gene fusion identification

SOAPfuse v1.26 (http://soap.genomics.org.cn/soapfuse.html) [17] was used for scanning of fusion RNAs using transcriptome data. Briefly, GRCh37.69.gtf.gz (*Homo sapiens*) was downloaded from Ensembl and used as gene annotation reference information (gtf). For cytoband information, the human genome (hg19, Reference 37) from UCSC, as well as the complete HGNC gene family dataset (HGNC), was used. The pipelines were tuned using Perl.

### Validation of fusion genes

Fusion genes were validated by reverse transcription-polymerase chain reaction (RT-PCR) amplification of fusion gene breakpoints, and Sanger sequencing. The PCR reactions were carried out for 4 min at 94 °C; 35 cycles of 40 s at 94 °C, 40 s at 55–58 °C and 40 s at 72 °C, and finally 7 min at 72 °C. The primer sequences are listed in Additional file 2: Table S4. PCR products were confirmed on a 2 % agarose gel, purified, and cloned into the pGEM-T easy vector (Promega, USA). The positive clones were selected for Sanger sequencing. *GAPDH* was used as a standard control.

### siRNA transfection

To suppress expression of *RNF43-SUPT4H1*, DLD-1 and HT29 cells were transiently transfected with siRNAs of the fusion transcript, and negative siRNA, in 6-well plates (2×10^5^ cells/well). The siRNAs sequences used against the *RNF43-SUPT4H1* fusion transcript variant 1 were candidate 1 in position 90 bp : 5′-CGA CAG CGC AAC AGA CUA U-3′ (sense) and 5′-AUA GUC UGU UGC GCU GUC G-3′ (antisense), and candidate 2 in position 97 bp: 5′-GCA ACA GAC UAU AGA CCA G-3′ (sense) and 5′-CUG GUC UAU AGU CUG UUG C-3′ (antisense) and negative siRNA were purchased from RNAi Co. (Bioneer, Korea). These siRNA candidates targeted fusion junction (Additional file 3: Figure S5). In each colorectal cancer cell line, 100 nM siRNA was treated using the RNAi MAX transfection reagent (Invitrogen), following the manufacturer’s instructions. The cells were harvested at 24, 48 and 72 h after transfection, and *RNF43-SUPT4H1* fusion transcript expression was analyzed by RT-PCR.

### MTT assay

Cell viability was assessed by tetrazolium salt reduction using the MTT [3-(4, 5-dimethylthiazol-2-yl)-2, 5-diphenyl tetrazolium bromide] assay (Sigma-Aldrich, USA). After siRNA transfection, the cells were incubated for 0, 24, 48, and 72 h before the addition of MTT substrate. MTT stock solution was added at a final concentration of 0.5 mg/ml, and cells were incubated at 37 °C for 1.25 h. MTT crystal was collected and dissolved by incubation with DMSO. Absorbance was measured by spectrophotometry at 540 nm wavelength.

### Access to data from this study

All RNA-seq data from this study are available for download through the NCBI Sequence Read Archive (SRA) (http://www.ncbi.nlm.nih.gov/sra), under accession number SRR2089755.

## Results

### Transcriptome sequencing and mapping

Five sets of quadruple-matched tissues (primary carcinomas, liver metastases, normal colon, and liver) were collected from Pusan National University Hospital. The clinical information for patients, whose samples were used in this study, is shown in Additional file 1: Table S1. All samples were subjected to high-throughput transcriptome sequencing. About 67.3–87.1 million raw reads from each samples were sequenced (Additional file 4: Table S2). After low-quality reads were filtered out, about 88.15–92.03 % reads were analyzed and mapped to the reference human genome Hg19. The average depth of coverage was >89 fold of that of the human transcriptome.

### Genes expression profiling

**Fig. 1:**
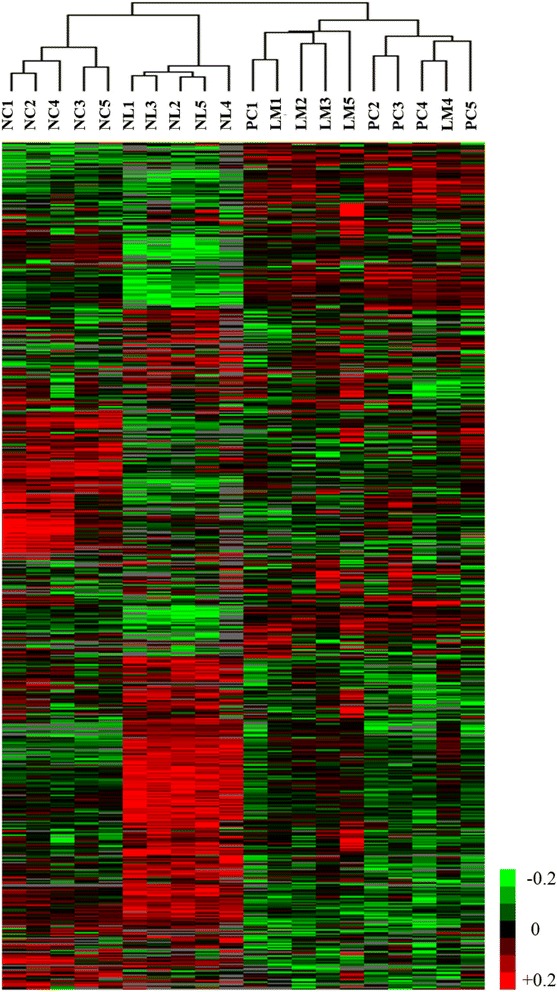
Hierarchical clustering of expression profiles. Data are presented in a matrix format, in which each row represents an individual gene and each column represents a different tissue sample. Red, high expression; green, low expression. NC, normal colon; PC, primary carcinoma; LM, liver metastases; NL, normal liver

The normalized expression level of each gene was expressed as Fragments Per Kilobase of Exon Per Million Fragments Mapped (FPKM). By setting a FPKM >1 threshold, we detected 56,268 reliable transcripts, which included the majority of the annotated human reference genes. We calculated the Pearson correlation coefficient to compare global gene expression between the samples. The correlation coefficients of primary carcinoma and liver metastases were higher compared to those of normal tissues (Additional file 5: Figure S2). In addition, unsupervised clustering analysis was performed. Genes with median of FPKM < 1.0 and coefficient of variation (CV) < 0.7 were excluded to remove genes noninformative for clustering. This resulted in a total of 7744 unique genes. The hierarchical clustering results showed that normal colon and liver were successfully distinguished from colorectal carcinoma, but primary carcinoma preferentially clustered with their matched liver metastases (Fig. 1). These results suggest a high concordance of gene expression in the primary carcinoma and liver metastases.

### Functional enrichment analysis of differentially expressed genes

The common 1895 DEGs in primary carcinoma and liver metastases, compared with normal colon, were detected. In order to investigate their roles in tumor development, we performed functional enrichment analysis of DEGs using the web-based tool DAVID [16]. The common 1895 DEGs were annotated in GO component, GO function, and GO process. Among the three GO categories, “cell cycle”, “cell division”, and “cellular process” were dominant (Table 1). These results suggested that common DEGs are related to tumor phenotype-associated processes, such as cell cycle regulation.

**Table 1:** Functional annotation of differentially expressed genes (1895 gene)

Term	Count	Percent	*P*-value	Benjamini
cell cycle	97	1.1	1.5E-36	7.8E-34
mitosis	55	0.6	7.7E-29	2.0E-26
cell division	61	0.7	3.0E-25	5.2E-23
kinetochore	25	0.3	2.0E-16	2.9E-14
centromere	17	0.2	3.6E-12	3.7E-10
nucleus	287	3.1	4.7E-12	4.1E-10
phosphoprotein	432	4.7	5.5E-11	4.1E-9
DNA replication	22	0.2	4.3E-10	2.8E-8
polymorphism	620	6.8	4.4E-9	2.6E-7
microtubule	30	0.3	1.2E-6	6.1E-5
cytoskeleton	58	0.6	1.7E-6	8.0E-5
acetylation	170	1.9	5.9E-6	2.6E-4
DNA damage	26	0.3	1.1E-5	4.2E-4
cadmium	6	0.1	1.1E-5	4.0E-4
ATP-binding	96	1.0	1.5E-5	5.3E-4
DNA repair	24	0.3	2.9E-5	9.4E-4
chromosomal protein	20	0.2	4.4E-5	1.4E-3
cell cycle control	9	0.1	6.5E-5	1.9E-3
ubl conjugation	49	0.5	1.2E-4	3.2E-3
metal-thiolate cluster	6	0.1	1.3E-4	3.4E-3
Fanconi anemia	6	0.1	2.0E-4	5.0E-3
acetylated amino end	15	0.2	2.1E-4	5.1E-3
chelation	5	0.1	2.8E-4	6.3E-3
Chromosome partition	8	0.1	4.5E-4	9.8E-3
nucleotide-binding	107	1.2	8.4E-4	1.7E-2
DNA condensation	5	0.1	1.7E-3	3.4E-2

We have also analyzed to select genes related to liver metastasis. We detected 694 genes differentially expressed between colorectal primary tumors and liver metastasis tumors (FDR < 0.05, fold change > 2). Of these genes, we selected 14 DEGs compared with normal colon (FDR < 0.05, fold > 2) and normal liver tissues (FDR < 0.05, fold >2) (Additional file 6: Table S3). Most of these genes are highly expressed in normal liver and their expression in liver metastase are lower.

### Detection of gene fusion events

**Fig. 2:**
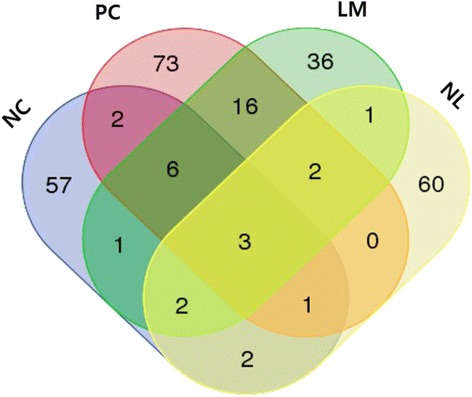
The Venn diagram for comparison of gene fusion events that are unique or shared in the 4 tissue types. NC, normal colon; PC, primary carcinoma; LM, liver metastases; NL, normal liver

To identify gene fusion events, SOAPfuse algorithm [17] was used. In this study, a total of 262 fusion events were found: normal colon, 74; primary carcinoma, 103; liver metastases, 67; normal liver, 71 fusion events. Gene fusion events were unique or shared among the four tissues types examined, as shown in Fig. 2. Within these gene fusion events, 73 and 36 cancer type-specific fusion events were found in the primary carcinoma and liver metastases, respectively. We focused on cancer type-specific events and gene fusions that are common in colorectal cancer, and selected fusion genes that arose due to in-frame fusions (Table 2). Most fusion partner genes were located on the same chromosome, while some were formed between genes on two different chromosomes.

**Table 2:** Summary of in-frame gene fusions

5′ Gene	Location	Direction	3′ Gene	Location	Direction	Chimera class	Cancer type	Patients no.^a^
GTF2E2	chr8:30510950	-	NGR1	chr8:32585467	+	intrachromosomal complex		1 (PC, LM)
TMEM66	chr8:29940363	-	NGR1	chr8:32453346	+	intrachromosomal complex	Identical patient	4 (PC, LM)
TNNC2	chr20:44452630	-	WFDC3	chr20:44404241	-	intrachromosomal complex		3 (PC, LM)
HEPHL1	chr11:93800911	+	PANX1	chr11:93862496	+	intrachromosomal_NG^b^		3 (PC, LM)
KIAA1984	chr9:139701518	+	C9orf86	chr9:139717977	+	intrachromosomal_NG	Common fusion	3 (LM); 4 (PC)
SLC39A1	chr1:153933048	-	CRTC2	chr1:153927642	-	intrachromosomal_NG		2 (PC); 4 (LM)
SCNN1A	chr12:6457893	-	TNFRSF1A	chr12:6443410	-	intrachromosomal_NG		3, 5 (PC); 1 (LM)
RUVBL1	chr3:127823674	-	LDHA	chr11:18424445	+	interchromosomal complex		2 (PC)
TPT1	chr13:45914237	-	YBX1	chr1:43149117	+	interchromosomal complex		3 (PC)
ZMYND8	chr20:45976600	-	SEPT9	chr17:75398141	+	interchromosomal complex		3 (PC)
SOLH	chr16:583998	+	NOC4L	chr12:132635526	+	interchromosomal complex		4 (PC)
CALR	chr19:13054619	+	EEF2	chr19:3981399	-	intrachromosomal complex		5 (PC)
DDX27	chr20:47855837	+	ZNFX1	chr20:47855592	-	intrachromosomal complex		3 (PC)
KIF3B	chr20:30865568	+	WFDC12	chr20:43752906	-	intrachromosomal complex		1 (PC)
GSK3B	chr3:119720893	-	POLQ	chr3:121155122	-	intrachromosomal complex		1 (PC)
CKLF	chr16:66597120	+	CMTM1	chr16:66611007	+	intrachromosomal_NG	Primary specific	2 (PC)
DUS4L	chr7:107217037	+	BCAP29	chr7:107221204	+	intrachromosomal_NG		1 (PC)
HSPE1	chr2:198367852	+	MOBKL3	chr2:198388348	+	intrachromosomal complex_NG		2 (PC)
PRIM1	chr12:57127931	-	NACA	chr12:57118307	-	intrachromosomal complex_NG		4 (PC)
RNF43	chr17:56494378	-	SUPT4H1	chr17:56428869	-	intrachromosomal_NG		4 (PC)
SLC10A3	chrX:153716020	-	UBL4A	chrX:153714672	-	intrachromosomal_NG		4 (PC)
UBA2	chr19:34957919	+	WTIP	chr19:34981281	+	intrachromosomal_NG		5 (PC)
ZNF606	chr19:58499575	-	C19orf18	chr19:58485571	-	intrachromosomal_NG		4 (PC)
HSP90AA1	chr14:102551123	-	NOP58	chr2:203165075	+	interchromosomal complex		1 (LM)
RPS15A	chr16:18794368	-	RPL0	chr8:99057311	-	interchromosomal complex		4 (LM)
FGG	chr4:155526082	-	ALB	chr4:74285288	+	intrachromosomal complex		2 (LM)
ACE2	chrX:15582147	-	PIR	chrX:15509432	-	intrachromosomal complex	Liver metastases specific	3 (LM)
OAF	chr11:120097705	+	POU2F3	chr11:120117158	+	intrachromosomal_NG		2 (LM)
SENP3	chr17:7474797	+	EIF4A1	chr17:7477578	+	intrachromosomal_NG		4 (LM)
TGIF2	chr20:35207369	+	C20orf24	chr20:35236118	+	intrachromosomal_NG		1 (LM)

^a^Patients No.: *PC* primary carcinoma, *LM* livermetastases

^b^ *NG* neighboring gene between the 5′ and 3′ fusion partner

### Validation of fusion genes

**Fig. 3:**
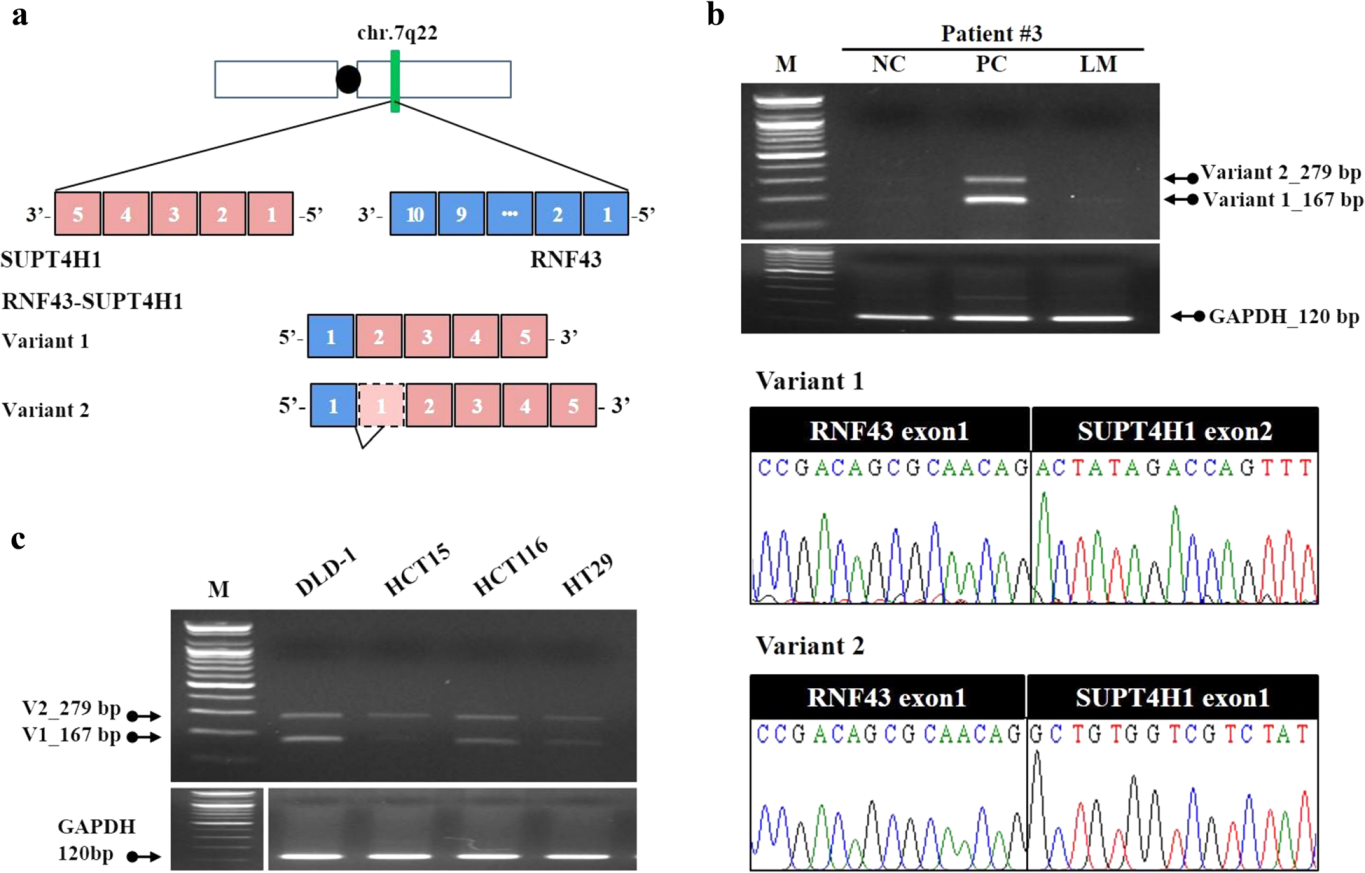
*RNF43-SUPT4H1* fusion in validation sets. **a** schematic of *RNF43, SUPT4H1* and the resulting *RNF43-SUPT4H1* fusion transcript. **b** PCR and Sanger sequencing validation of the positive fusion samples in validation sets. NC, normal colon; PC, primary carcinoma; LM, liver metastases. **c** *RNF43-SUPT4H1* fusion screening in colorectal cancer cell lines

In order to experimentally confirm the gene fusions identified by RNA-Seq, three fusion transcripts were selected for validation by RT-PCR. We chose three cases of fusion events, representing inter-chromosomal and intra-chromosomal complex rearrangements, and read-through transcription. A primer pair was designed to coordinate with the first exon of *RNF43* and the exon junction, as well as the second and third exons of *SUPT4H1* (Fig. 3a). The results confirmed the fusion event in the primary carcinoma, and the fusion junction was confirmed by Sanger sequencing (Fig. 3b). Also, we found an alternative fusion transcript in the primary carcinoma, which contained a part of the first exon of the *SUPT4H1* gene. In addition, *ZMYND8-SEPT9* and *ACE2-PIR* fusion transcripts were also successfully amplified by RT-PCR, and these fusion junctions were confirmed by Sanger sequencing (Additional file 7: Figure S3). These results confirmed fusion events in the sample, consistent with results of RNA-seq analysis.

To confirm the frequency of occurrence of the *RNF43-SUPT4H1* fusion, we screened for the expression of the fusion transcript in ten paired samples (Additional file 8: Figure S4). The *RNF43-SUPT4H1* fusion transcripts were found to occur frequently, and exhibit cancer-specific expression patterns. In addition, we screened 4 colorectal cancer cell lines using fusion-specific PCR primers, for additional confirmation of frequency of the *RNF43-SUPT4H1* fusion. *RNF43-SUPT4H1* fusion transcripts were identified in all four colorectal cancer cell lines (Fig. 3c).

### Functional analysis of the RNF43-SUPT4H1 fusion gene

**Fig. 4:**
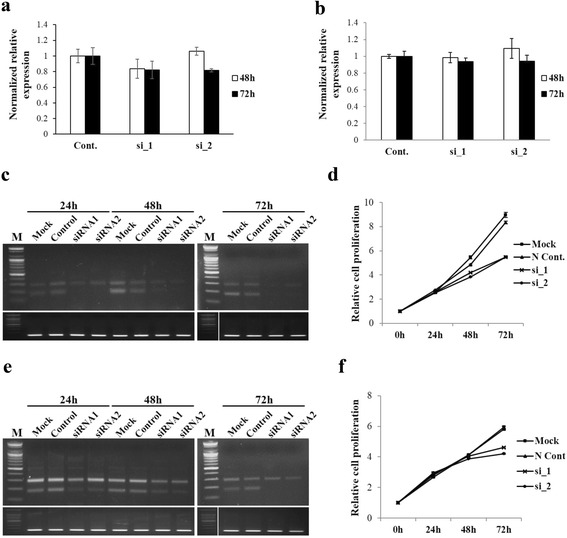
Knockdown of RNF43-SUPT4H1 fusion transcript results in decreased cell proliferation. Quantitative RT-PCR of original RNF43 (**a**) and *SUPT4H1* (**b**) gene in the DLD-1 cell line after transfection of siRNA targeting the *RNF43-SUPT4H1* fusion transcript. **c** and **e**, RT-PCR of *RNF43-SUPT4H1* fusion transcript in the DLD-1 and HT29 cell line after siRNA treatment. **d** and **f**, Knockdown of *RNF43-SUPT4H1* fusion transcript decreased cell proliferation in the DLD-1 and HT29 cell lines

In order to investigate the role of the *RNF43-SUPT4H1* fusion transcript in colorectal cancer cell growth, the expression of fusion transcript variant 1 was downregulated in colorectal cancer cell lines, DLD-1 and HT29. We synthesized two candidate siRNAs against the fusion transcript. Endogenous expression of fusion transcript variant 1 was successfully inhibited in the DLD-1 and HT29 cell lines by both *RNF43-SUPT4H1* candidate siRNAs (Fig. 4c, f); however, siRNA transfection had no effect on the expression of the original *RNF43* and *SUPT4H1* gene (Fig. 4a, b). Knockdown of fusion transcript variant 1 resulted in a significant decrease in cell proliferation at 48 and 72 h after transfection in the DLD-1 cell line (Fig. 4d). In the HT29 cell line, cell proliferation similarly decreased at 72 h after transfection (Fig. 4f). These results suggest that the *RNF43-SUPT4H1* fusion transcript has a positive effect on cell growth in colorectal cancer.

## Discussion

In this study, we performed transcriptome analysis using RNA-seq, to compare the gene expression profiles of primary colorectal carcinoma and liver metastases. Our results revealed high concordance of gene expression between the primary carcinoma and liver metastases. Interestingly, we found that fusion transcripts are expressed differentially between the primary colorectal cancer and liver metastases. Our results also suggest that the fusion genes investigated may serve as potential new targets for primary colorectal carcinoma.

A recent study reported high genomic concordance between primary carcinoma and metastases in colorectal cancer [18, 19]. In our study, the result of unsupervised clustering was in agreement with that of previous reports. These results suggest that primary tumor and metastases may share molecular profiles at different regions. Because cancer cells that leave the primary tumor can seed metastases in distant organs [19, 20]. However, each patient clustering showed different expression patterns between primary cancers and their metastases (Additional file 9: Figure S1). In addition, we identified 14 statistically significant genes associated with liver metastases. We will further investigate the roles of DEGs in colon cancer metastasis.

In this study, we focused on the structure of the transcriptome and analyzed cancer type-specific fusion transcripts. Gene fusion events that result in genomic aberrations or transcription-mediated chimeric oncogenes are known to be involved in cancer development and progression. Fusion transcripts have been found in various cancers, including *EML4-ALK* in lung [21], *ETV6-NTRK3* in breast [22], and translocation of genes in the ETS family in prostate cancer [23]. The expression of these fusion transcripts influences cell growth, colony formation, migration, and invasion, which often results from the production of functional proteins [7]. In colorectal cancer, however, fusion transcripts are not commonly reported [24]. Investigating cancer type-specific gene fusion is useful for understanding the complexity of the cancer genome, and studying colorectal cancer development [14]. In the present study, gene fusion events in primary colorectal carcinoma and liver metastases tissues were detected using RNA-seq technique. A total of 30 in-frame fusion transcripts were identified in primary carcinoma and liver metastases. Among these fusion transcripts, *GTF2E2-NRG1*, *TMEM66-NRG1*, *TNNC2-WFDC,* and *HEPHL1-PANX1* fusion transcripts were found in both primary carcinoma and liver metastases from the same patient. It is considered that these fusion transcripts, with the exception of the *HEPHL1-PANX1* gene fusion, were generated due to genomic aberrations, e.g., inversion or deletion. However, common cancer type-specific fusion transcripts are generated by transcription-mediated mechanisms, including read-through and trans-splicing, allowing for high concordance between the genomes of primary tumors and metastases. The *ZMYND8-SEPT9* fusion transcript, which arises due to a fusion event involving genes on different chromosomes, is only present in primary carcinoma (Additional file 7: Figure S3A). Therefore, we suggest that the cancer type-specific fusion transcripts enable differentiation between primary carcinoma and liver metastases at the transcriptome level, regardless of genomic variation.

The Cancer Genome Atlas (TCGA) has recently reported genomic aberrations of colorectal cancer, using high-throughput sequencing [13]. The TCGA study, which focused on translocation–mediated gene fusions, reported 18 interchromosomal translocation and in-frame events. Gene fusion events may additionally occur due to genomic rearrangements. Transcription-mediated gene fusions show high frequency, and recurrent functional gene fusions are suggested as candidate biomarkers and potential therapeutic targets. We detected not only genomic rearrangement-mediated gene fusion, but also transcription-mediated gene fusion events (Table 2). Among these fusion genes, the *CNN1A-TNFRSF1A* fusion transcript, which is translated into fusion protein, has been reported in breast cancer [25]. Furthermore, *DUS4L–BCAP29* fusion transcript has been reported in gastric cancer, which encodes a functional protein that is involved in cell proliferation [26]. We report, for the first time, that knockdown of the *RNF43-SUPT4H1* fusion transcript reduces cell proliferation in live cells suggesting this fusion transcript plays a role in cancer cell growth. Therefore, we suggest that these fusion transcripts may serve as potential biomarker candidates and therapeutic targets.

The genomic loci of the *RNF43* and *SUPT4H1* genes are adjacent to each other, and the *RNF43-SUPT4H1* fusion transcript is found to occur frequently. As a result, the *RNF43-SUPT4H1* fusion transcript was categorized as a read-through chimera. This fusion transcript was detected in cancer tissues only (Fig. 3 and Additional file 8: Figure S4). We therefore hypothesized that *RNF43-SUPT4H1* fusion transcript acts as an oncogene, and confirmed this function (Fig. 4). *RNF43* encodes the ring finger protein 43 that is involved in cell growth, and is upregulated in human colon cancer [27]. *SUPT4H1* encodes the transcription elongation factor SPT4, which regulates mRNA processing and transcription elongation [28]. We speculate that the *RNF43-SUPT4H1* fusion transcript is activated in colorectal cancer, affecting the expression of other genes. Future studies should focus on investigating the function of cancer type-specific fusion transcripts and developing methods for distinguishing between primary carcinoma and liver metastases.

## Conclusion

This study presents the expression profiles of primary carcinoma and matched liver metastases in colorectal cancer, and reports several fusion transcripts associated with these tumor types. Although the gene expression profiles of primary carcinoma and matched liver metastases were similar, we identified cancer type-specific fusion transcripts that may be useful for distinguishing between primary carcinoma and liver metastases. These findings may be valuable for further studies of colorectal cancer metastasis, biomarker discovery and target identification in therapeutic drug discovery.

## Abbreviations

CRC, colorectal cancers; CV, coefficient of variation; DEG, differentially expressed gene; FDR, false discovery rate; FPKM, fragments per kb per million fragments; GO, gene ontology; LM, colon-liver metastases; MTT, 3-(4, 5–20 dimethylthiazol-2-yl)-2, 5-diphenyl tetrazolium bromide; NC, normal colon; NL, normal liver; PC, primary colon carcinoma; RNA-seq, RNA sequencing; RT-PCR, reverse transcription-polymerase chain reaction; siRNA, small interfering RNA; SRA, sequence read archive; TCGA, the cancer genome atlas

## Additional file

Additional file 1: Table S1. Clinical information of patients used in this study. Additional file 2: Table S4. Primer information for gene fusion validation. Additional file 3: Figure S5. RNF43-SUPT4H1 fusion transcript variant 1 targeted siRNA candidates. siRNA candidates were designed to including fusion junction. Red arrow was fusion junction, and each under bars were siRNA candidates. Additional file 4: Table S2. Summary of statistical data for whole-transcriptome sequencing data used in this study. Additional file 5: Figure S2. The scatter plot for global expression between samples; Pearson correlation coefficient is shown. Additional file 6: Table S3. Genes (*n*=14) associated with liver metastases as compared to primary tumors. Additional file 7: Figure S3. Fusion transcripts in validation sets. (A) Gene fusion between ZMYND8 and SEPT9 gene by interchromosomal complex. (B) Gene fusion between ACE2 and PIR gene by intrachromosomal complex. Fusion junction was red arrow, and validation of fusion transcript by RT-PCR and Sanger sequencing in patient #3. Prediction of fusion protein was analyzed by conserved domain database. Additional file 8: Figure S4. RNF43-SUPT4H1 fusion transcript frequency in 10 paired colorectal cancer and normal tissues. M, size marker; N, normal; T, tumor. Additional file 9: Figure S1. Hierarchical clustering of expression profiles. Data are pre-sented in a matrix format, in which each row represents an indi-vidual gene and each column represents a different tissue sam-ple. Each cell in the matrix represents the expression level of a gene feature in an individual tissue sample. Red, high expres-sion; green, low expression. N, normal colon; C, primary carci-noma; LM, liver metastases; NL, normal liver. (PPTX 1128 kb)
