# Supplementary material for: Transcriptome analysis of paired primary colorectal carcinoma and liver metastases reveals fusion transcripts and similar gene expression profiles in primary carcinoma and liver metastases
Source: BMC Cancer. 2016 Jul 26;16:539. doi: 10.1186/s12885-016-2596-3 (PMC4962348; doi:10.1186/s12885-016-2596-3)
Supplement: Additional file 1: Table S1. — Clinical information of patients used in this study. [file 12885_2016_2596_MOESM1_ESM.pptx]

## Slide 1
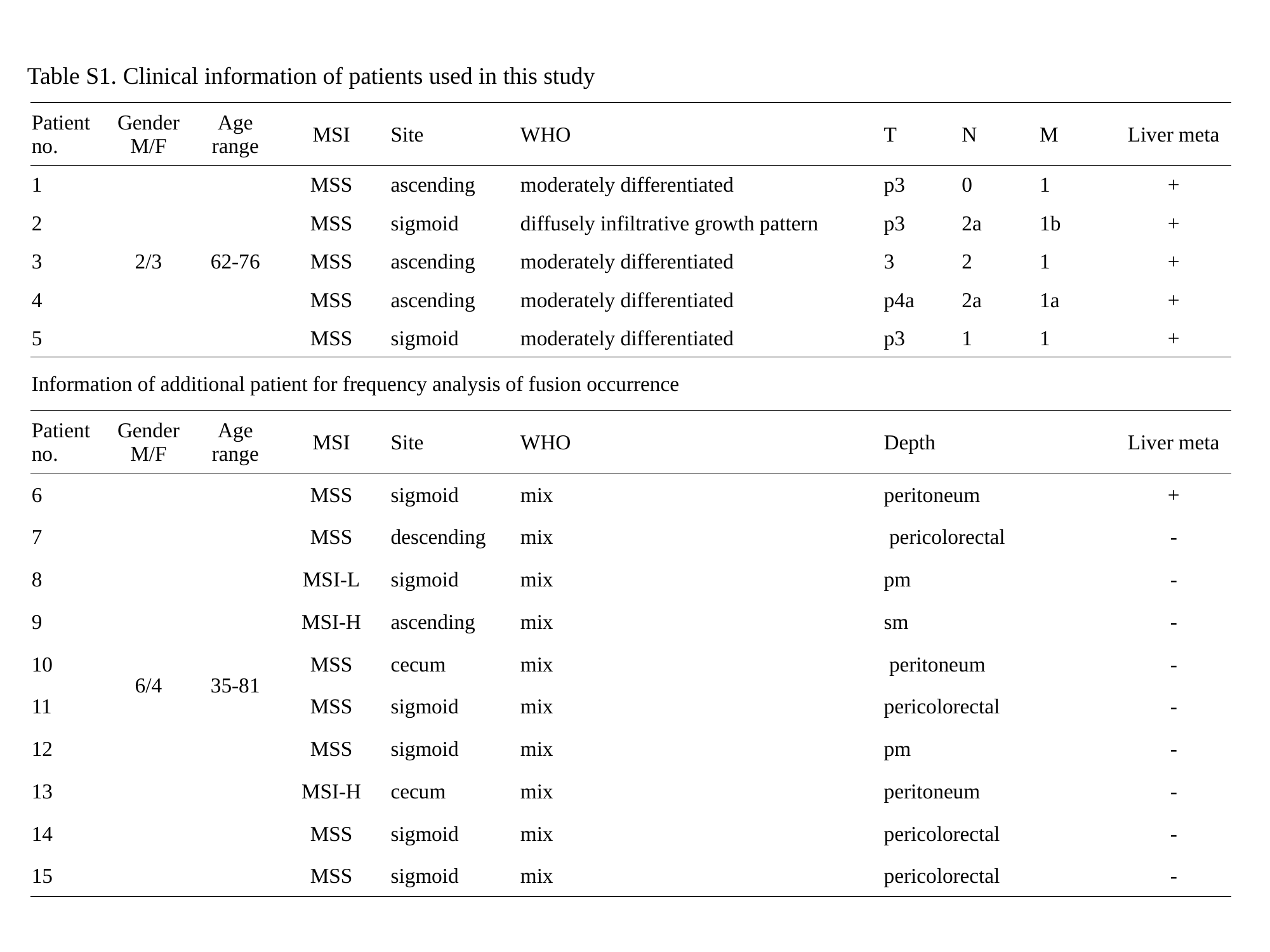

Table S1. Clinical information of patients used in this study
| Patient no. | Gender M/F | Age range | MSI | Site | WHO | T | N | M | Liver meta |
| --- | --- | --- | --- | --- | --- | --- | --- | --- | --- |
| 1 | 2/3 | 62-76 | MSS | ascending | moderately differentiated | p3 | 0 | 1 | + |
| 2 | | | MSS | sigmoid | diffusely infiltrative growth pattern | p3 | 2a | 1b | + |
| 3 | | | MSS | ascending | moderately differentiated | 3 | 2 | 1 | + |
| 4 | | | MSS | ascending | moderately differentiated | p4a | 2a | 1a | + |
| 5 | | | MSS | sigmoid | moderately differentiated | p3 | 1 | 1 | + |
| Information of additional patient for frequency analysis of fusion occurrence | | | | | | | | | |
| Patient no. | Gender M/F | Age range | MSI | Site | WHO | Depth | | | Liver meta |
| 6 | 6/4 | 35-81 | MSS | sigmoid | mix | peritoneum | | | + |
| 7 | | | MSS | descending | mix | pericolorectal | | | - |
| 8 | | | MSI-L | sigmoid | mix | pm | | | - |
| 9 | | | MSI-H | ascending | mix | sm | | | - |
| 10 | | | MSS | cecum | mix | peritoneum | | | - |
| 11 | | | MSS | sigmoid | mix | pericolorectal | | | - |
| 12 | | | MSS | sigmoid | mix | pm | | | - |
| 13 | | | MSI-H | cecum | mix | peritoneum | | | - |
| 14 | | | MSS | sigmoid | mix | pericolorectal | | | - |
| 15 | | | MSS | sigmoid | mix | pericolorectal | | | - |
