# Supplementary material for: Transcriptome analysis of paired primary colorectal carcinoma and liver metastases reveals fusion transcripts and similar gene expression profiles in primary carcinoma and liver metastases
Source: BMC Cancer. 2016 Jul 26;16:539. doi: 10.1186/s12885-016-2596-3 (PMC4962348; doi:10.1186/s12885-016-2596-3)
Supplement: Additional file 2: Table S4. — Primer information for gene fusion validation. [file 12885_2016_2596_MOESM2_ESM.pptx]

## Slide 1
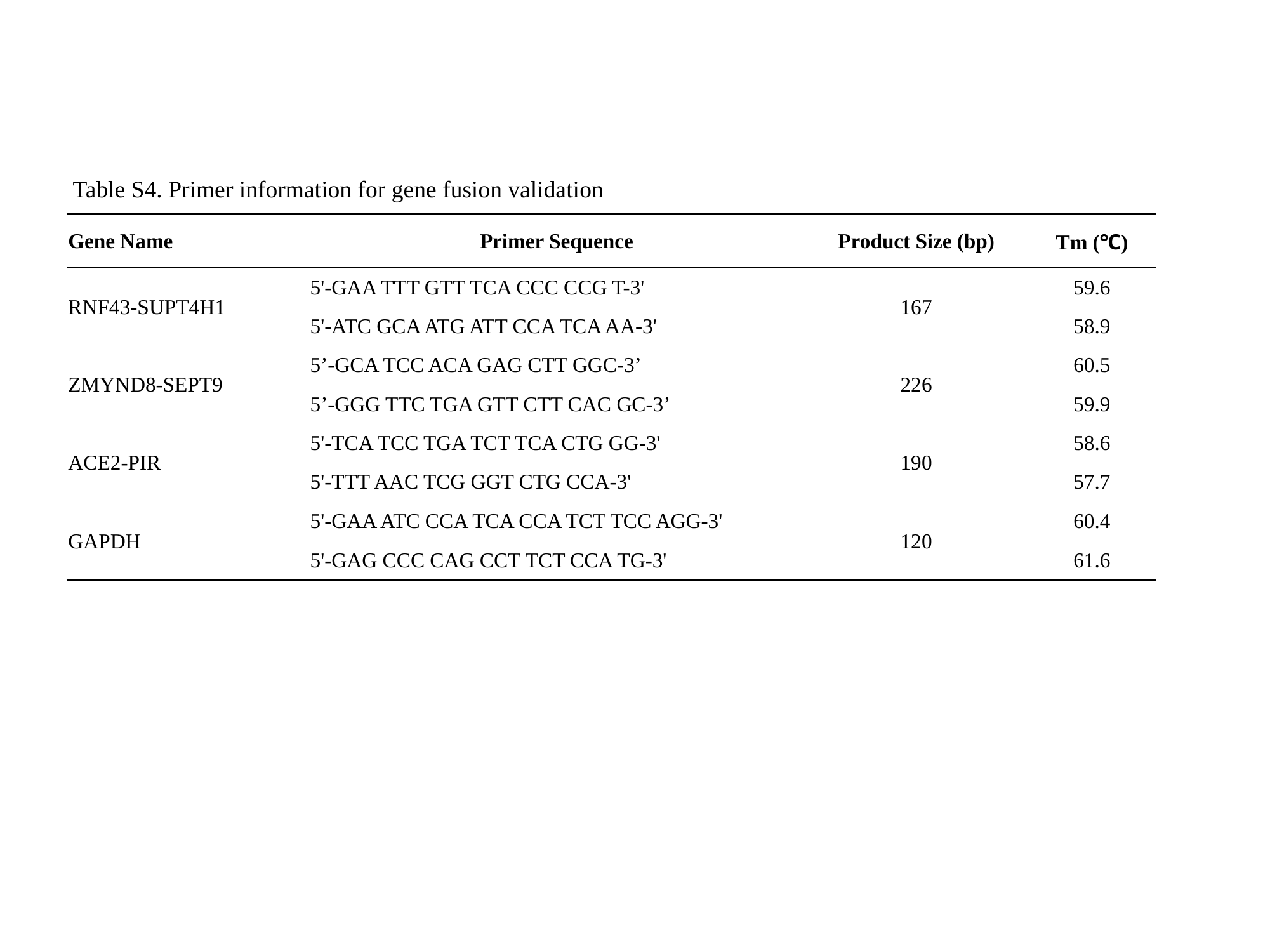

Table S4. Primer information for gene fusion validation
| Gene Name | Primer Sequence | Product Size (bp) | Tm (℃) |
| --- | --- | --- | --- |
| RNF43-SUPT4H1 | 5'-GAA TTT GTT TCA CCC CCG T-3' | 167 | 59.6 |
| | 5'-ATC GCA ATG ATT CCA TCA AA-3' | | 58.9 |
| ZMYND8-SEPT9 | 5’-GCA TCC ACA GAG CTT GGC-3’ | 226 | 60.5 |
| | 5’-GGG TTC TGA GTT CTT CAC GC-3’ | | 59.9 |
| ACE2-PIR | 5'-TCA TCC TGA TCT TCA CTG GG-3' | 190 | 58.6 |
| | 5'-TTT AAC TCG GGT CTG CCA-3' | | 57.7 |
| GAPDH | 5'-GAA ATC CCA TCA CCA TCT TCC AGG-3' | 120 | 60.4 |
| | 5'-GAG CCC CAG CCT TCT CCA TG-3' | | 61.6 |
