# Supplementary material for: Transcriptome analysis of paired primary colorectal carcinoma and liver metastases reveals fusion transcripts and similar gene expression profiles in primary carcinoma and liver metastases
Source: BMC Cancer. 2016 Jul 26;16:539. doi: 10.1186/s12885-016-2596-3 (PMC4962348; doi:10.1186/s12885-016-2596-3)
Supplement: Additional file 3: Figure S5. — RNF43-SUPT4H1 fusion transcript variant 1 targeted siRNA candidates. siRNA candidates were designed to including fusion junction. Red arrow was fusion junction, and each under bars were siRNA candidates. [file 12885_2016_2596_MOESM3_ESM.pptx]

## Slide 1
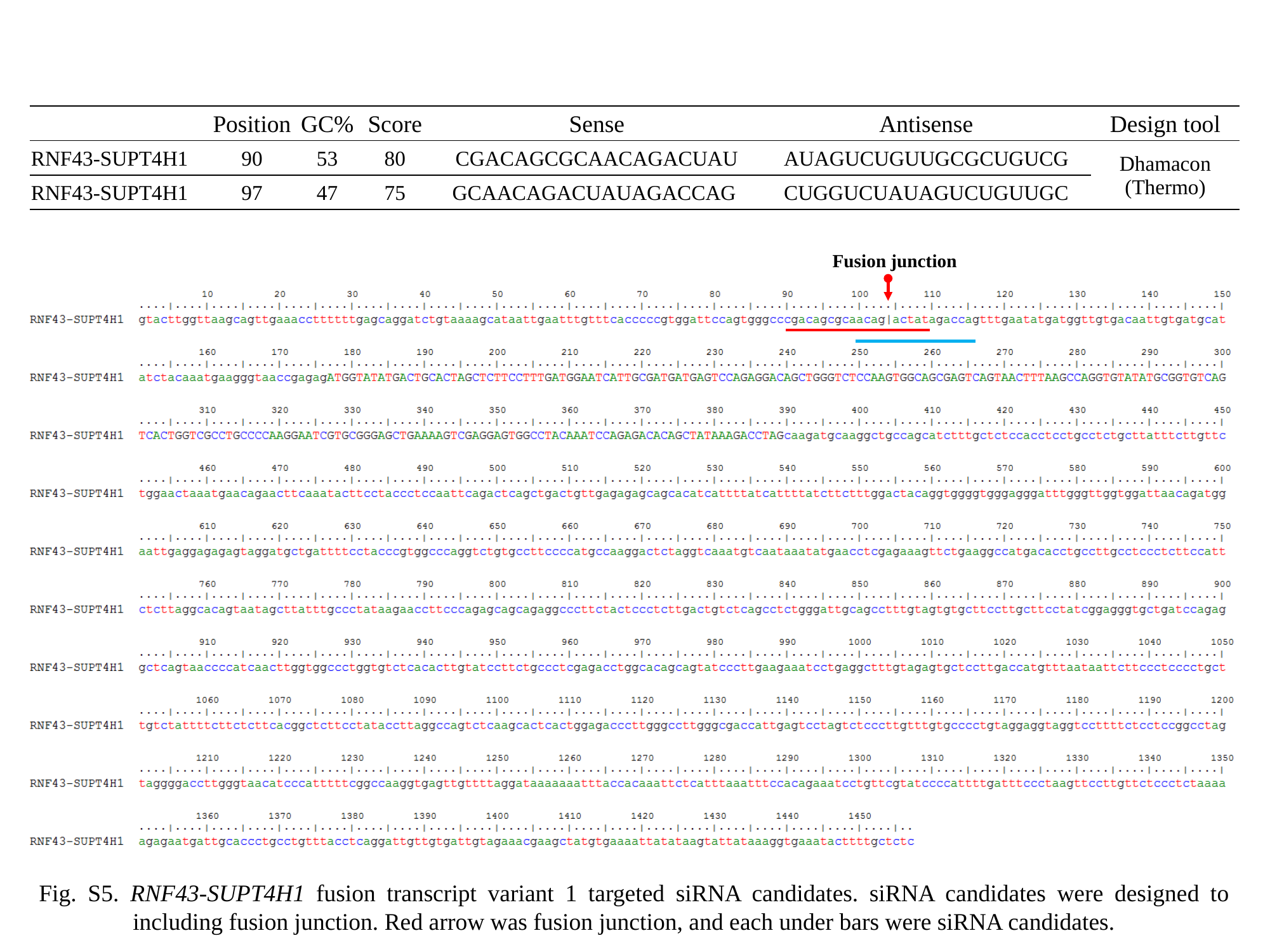

| | Position | GC% | Score | Sense | Antisense | Design tool |
| --- | --- | --- | --- | --- | --- | --- |
| RNF43-SUPT4H1 | 90 | 53 | 80 | CGACAGCGCAACAGACUAU | AUAGUCUGUUGCGCUGUCG | Dhamacon (Thermo) |
| RNF43-SUPT4H1 | 97 | 47 | 75 | GCAACAGACUAUAGACCAG | CUGGUCUAUAGUCUGUUGC | |
Fusion junction
Fig. S5. RNF43-SUPT4H1 fusion transcript variant 1 targeted siRNA candidates. siRNA candidates were designed to including fusion junction. Red arrow was fusion junction, and each under bars were siRNA candidates.
