# Supplementary material for: Transcriptome analysis of paired primary colorectal carcinoma and liver metastases reveals fusion transcripts and similar gene expression profiles in primary carcinoma and liver metastases
Source: BMC Cancer. 2016 Jul 26;16:539. doi: 10.1186/s12885-016-2596-3 (PMC4962348; doi:10.1186/s12885-016-2596-3)
Supplement: Additional file 4: Table S2. — Summary of statistical data for whole-transcriptome sequencing data used in this study. [file 12885_2016_2596_MOESM4_ESM.pptx]

## Slide 1
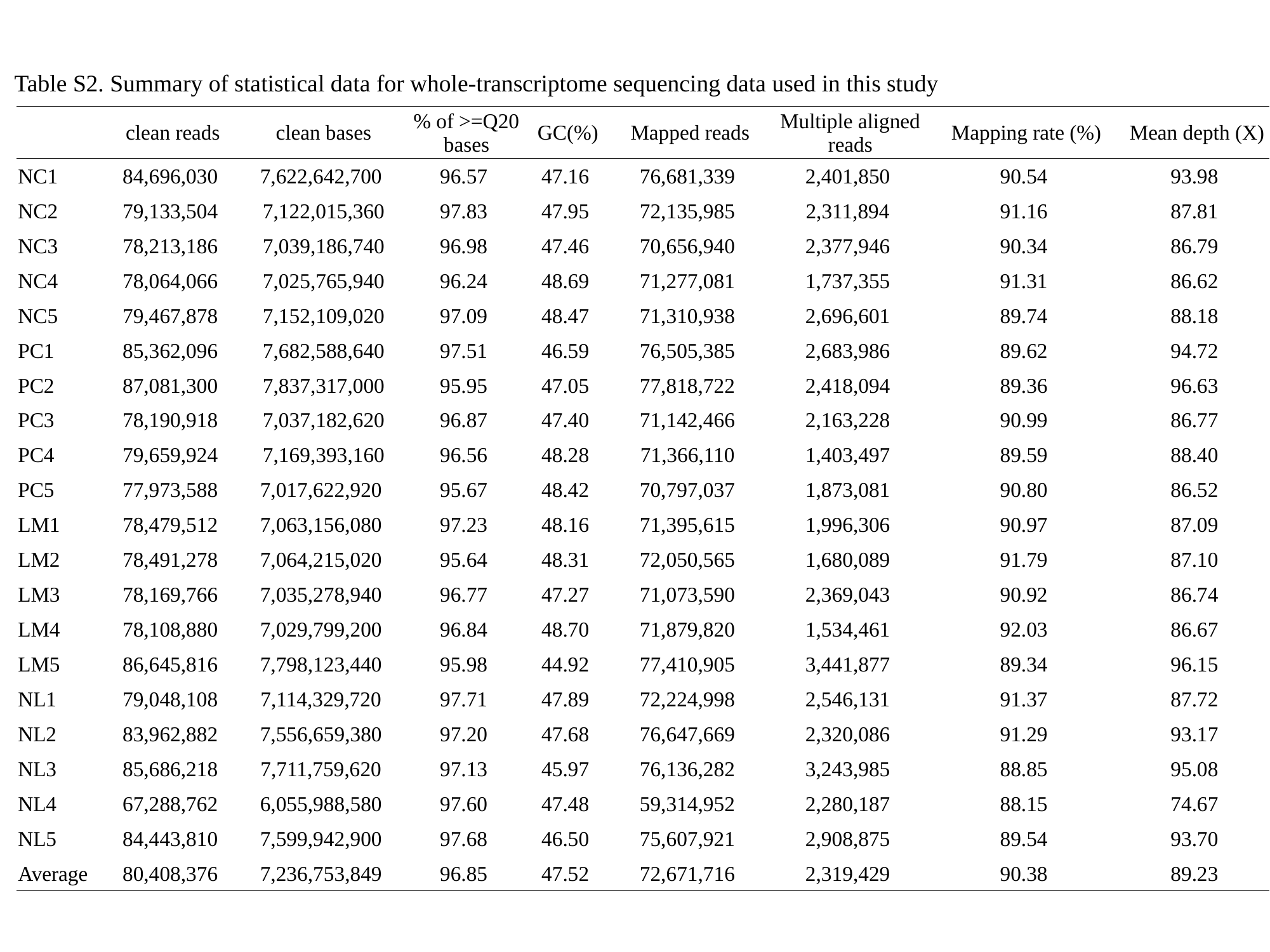

Table S2. Summary of statistical data for whole-transcriptome sequencing data used in this study
| | clean reads | clean bases | % of >=Q20 bases | GC(%) | Mapped reads | Multiple aligned reads | Mapping rate (%) | Mean depth (X) |
| --- | --- | --- | --- | --- | --- | --- | --- | --- |
| NC1 | 84,696,030 | 7,622,642,700 | 96.57 | 47.16 | 76,681,339 | 2,401,850 | 90.54 | 93.98 |
| NC2 | 79,133,504 | 7,122,015,360 | 97.83 | 47.95 | 72,135,985 | 2,311,894 | 91.16 | 87.81 |
| NC3 | 78,213,186 | 7,039,186,740 | 96.98 | 47.46 | 70,656,940 | 2,377,946 | 90.34 | 86.79 |
| NC4 | 78,064,066 | 7,025,765,940 | 96.24 | 48.69 | 71,277,081 | 1,737,355 | 91.31 | 86.62 |
| NC5 | 79,467,878 | 7,152,109,020 | 97.09 | 48.47 | 71,310,938 | 2,696,601 | 89.74 | 88.18 |
| PC1 | 85,362,096 | 7,682,588,640 | 97.51 | 46.59 | 76,505,385 | 2,683,986 | 89.62 | 94.72 |
| PC2 | 87,081,300 | 7,837,317,000 | 95.95 | 47.05 | 77,818,722 | 2,418,094 | 89.36 | 96.63 |
| PC3 | 78,190,918 | 7,037,182,620 | 96.87 | 47.40 | 71,142,466 | 2,163,228 | 90.99 | 86.77 |
| PC4 | 79,659,924 | 7,169,393,160 | 96.56 | 48.28 | 71,366,110 | 1,403,497 | 89.59 | 88.40 |
| PC5 | 77,973,588 | 7,017,622,920 | 95.67 | 48.42 | 70,797,037 | 1,873,081 | 90.80 | 86.52 |
| LM1 | 78,479,512 | 7,063,156,080 | 97.23 | 48.16 | 71,395,615 | 1,996,306 | 90.97 | 87.09 |
| LM2 | 78,491,278 | 7,064,215,020 | 95.64 | 48.31 | 72,050,565 | 1,680,089 | 91.79 | 87.10 |
| LM3 | 78,169,766 | 7,035,278,940 | 96.77 | 47.27 | 71,073,590 | 2,369,043 | 90.92 | 86.74 |
| LM4 | 78,108,880 | 7,029,799,200 | 96.84 | 48.70 | 71,879,820 | 1,534,461 | 92.03 | 86.67 |
| LM5 | 86,645,816 | 7,798,123,440 | 95.98 | 44.92 | 77,410,905 | 3,441,877 | 89.34 | 96.15 |
| NL1 | 79,048,108 | 7,114,329,720 | 97.71 | 47.89 | 72,224,998 | 2,546,131 | 91.37 | 87.72 |
| NL2 | 83,962,882 | 7,556,659,380 | 97.20 | 47.68 | 76,647,669 | 2,320,086 | 91.29 | 93.17 |
| NL3 | 85,686,218 | 7,711,759,620 | 97.13 | 45.97 | 76,136,282 | 3,243,985 | 88.85 | 95.08 |
| NL4 | 67,288,762 | 6,055,988,580 | 97.60 | 47.48 | 59,314,952 | 2,280,187 | 88.15 | 74.67 |
| NL5 | 84,443,810 | 7,599,942,900 | 97.68 | 46.50 | 75,607,921 | 2,908,875 | 89.54 | 93.70 |
| Average | 80,408,376 | 7,236,753,849 | 96.85 | 47.52 | 72,671,716 | 2,319,429 | 90.38 | 89.23 |
