# Supplementary material for: Transcriptome analysis of paired primary colorectal carcinoma and liver metastases reveals fusion transcripts and similar gene expression profiles in primary carcinoma and liver metastases
Source: BMC Cancer. 2016 Jul 26;16:539. doi: 10.1186/s12885-016-2596-3 (PMC4962348; doi:10.1186/s12885-016-2596-3)
Supplement: Additional file 5: Figure S2. — The scatter plot for global expression between samples; Pearson correlation coefficient is shown. [file 12885_2016_2596_MOESM5_ESM.pptx]

## Slide 1
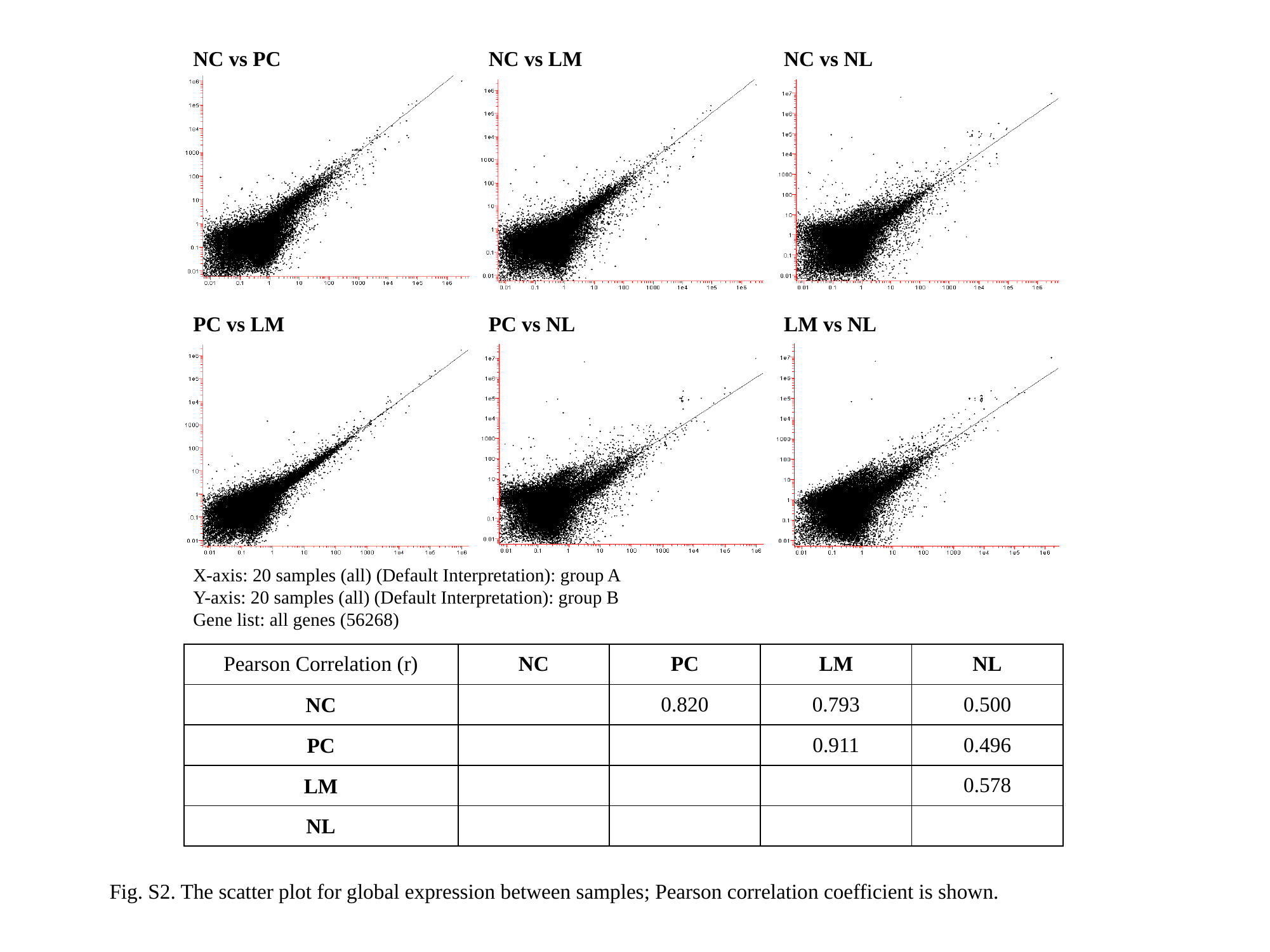

NC vs PC
NC vs LM
NC vs NL
PC vs LM
PC vs NL
LM vs NL
X-axis: 20 samples (all) (Default Interpretation): group A
Y-axis: 20 samples (all) (Default Interpretation): group B
Gene list: all genes (56268)
| Pearson Correlation (r) | NC | PC | LM | NL |
| --- | --- | --- | --- | --- |
| NC | | 0.820 | 0.793 | 0.500 |
| PC | | | 0.911 | 0.496 |
| LM | | | | 0.578 |
| NL | | | | |
Fig. S2. The scatter plot for global expression between samples; Pearson correlation coefficient is shown.
