# Supplementary material for: Transcriptome analysis of paired primary colorectal carcinoma and liver metastases reveals fusion transcripts and similar gene expression profiles in primary carcinoma and liver metastases
Source: BMC Cancer. 2016 Jul 26;16:539. doi: 10.1186/s12885-016-2596-3 (PMC4962348; doi:10.1186/s12885-016-2596-3)
Supplement: Additional file 6: Table S3. — Genes (n=14) associated with liver metastases as compared to primary tumors. [file 12885_2016_2596_MOESM6_ESM.pptx]

## Slide 1
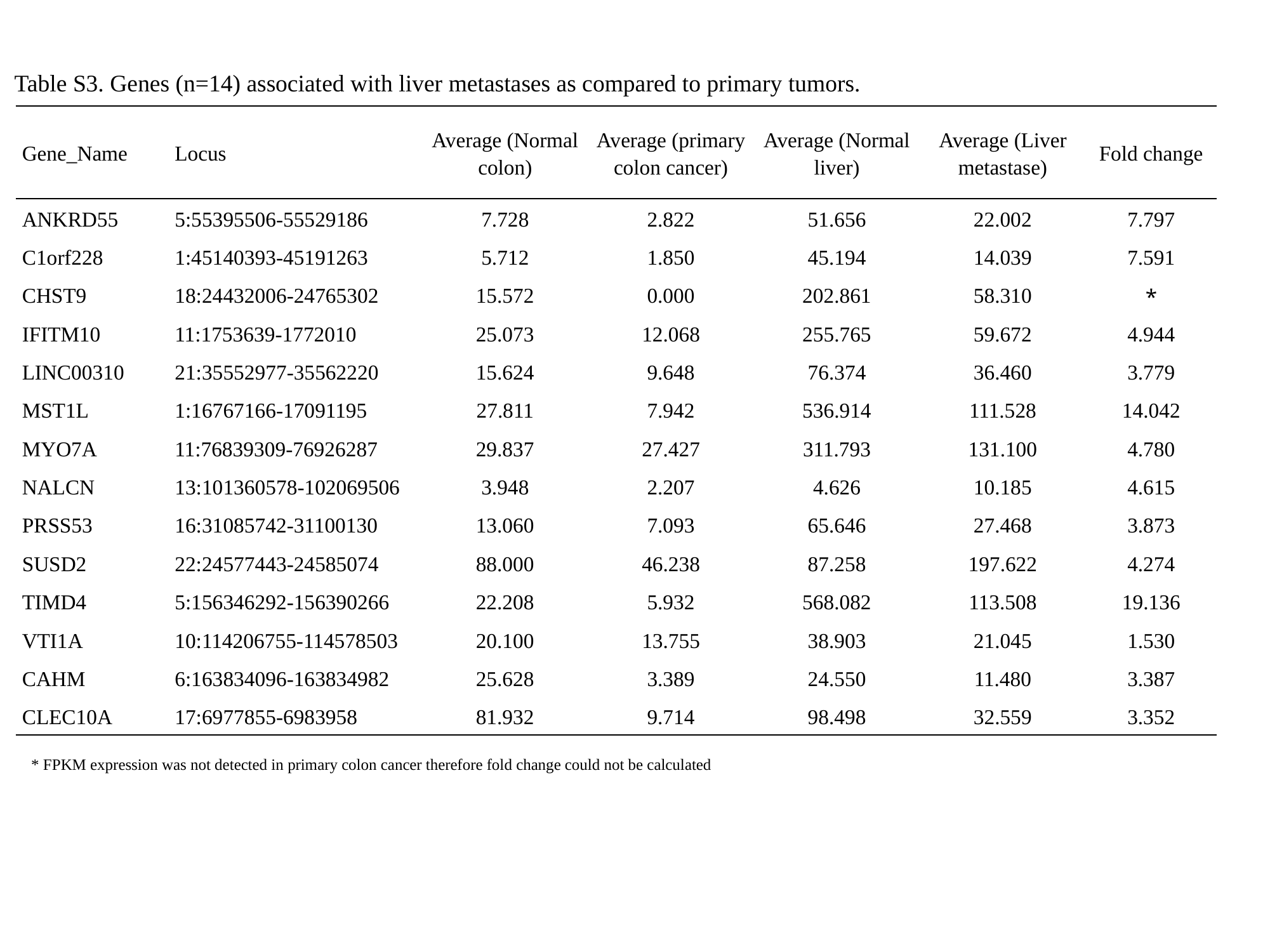

Table S3. Genes (n=14) associated with liver metastases as compared to primary tumors.
| Gene\_Name | Locus | Average (Normal colon) | Average (primary colon cancer) | Average (Normal liver) | Average (Liver metastase) | Fold change |
| --- | --- | --- | --- | --- | --- | --- |
| ANKRD55 | 5:55395506-55529186 | 7.728 | 2.822 | 51.656 | 22.002 | 7.797 |
| C1orf228 | 1:45140393-45191263 | 5.712 | 1.850 | 45.194 | 14.039 | 7.591 |
| CHST9 | 18:24432006-24765302 | 15.572 | 0.000 | 202.861 | 58.310 | \* |
| IFITM10 | 11:1753639-1772010 | 25.073 | 12.068 | 255.765 | 59.672 | 4.944 |
| LINC00310 | 21:35552977-35562220 | 15.624 | 9.648 | 76.374 | 36.460 | 3.779 |
| MST1L | 1:16767166-17091195 | 27.811 | 7.942 | 536.914 | 111.528 | 14.042 |
| MYO7A | 11:76839309-76926287 | 29.837 | 27.427 | 311.793 | 131.100 | 4.780 |
| NALCN | 13:101360578-102069506 | 3.948 | 2.207 | 4.626 | 10.185 | 4.615 |
| PRSS53 | 16:31085742-31100130 | 13.060 | 7.093 | 65.646 | 27.468 | 3.873 |
| SUSD2 | 22:24577443-24585074 | 88.000 | 46.238 | 87.258 | 197.622 | 4.274 |
| TIMD4 | 5:156346292-156390266 | 22.208 | 5.932 | 568.082 | 113.508 | 19.136 |
| VTI1A | 10:114206755-114578503 | 20.100 | 13.755 | 38.903 | 21.045 | 1.530 |
| CAHM | 6:163834096-163834982 | 25.628 | 3.389 | 24.550 | 11.480 | 3.387 |
| CLEC10A | 17:6977855-6983958 | 81.932 | 9.714 | 98.498 | 32.559 | 3.352 |
* FPKM expression was not detected in primary colon cancer therefore fold change could not be calculated
