# Supplementary material for: Transcriptome analysis of paired primary colorectal carcinoma and liver metastases reveals fusion transcripts and similar gene expression profiles in primary carcinoma and liver metastases
Source: BMC Cancer. 2016 Jul 26;16:539. doi: 10.1186/s12885-016-2596-3 (PMC4962348; doi:10.1186/s12885-016-2596-3)
Supplement: Additional file 7: Figure S3. — Fusion transcripts in validation sets. (A) Gene fusion between ZMYND8 and SEPT9 gene by interchromosomal complex. (B) Gene fusion between ACE2 and PIR gene by intrachromosomal complex. Fusion junction was red arrow, and validation of fusion transcript by RT-PCR and Sanger sequencing in patient #3. Prediction of fusion protein was analyzed by conserved domain database. [file 12885_2016_2596_MOESM7_ESM.pptx]

## Slide 1
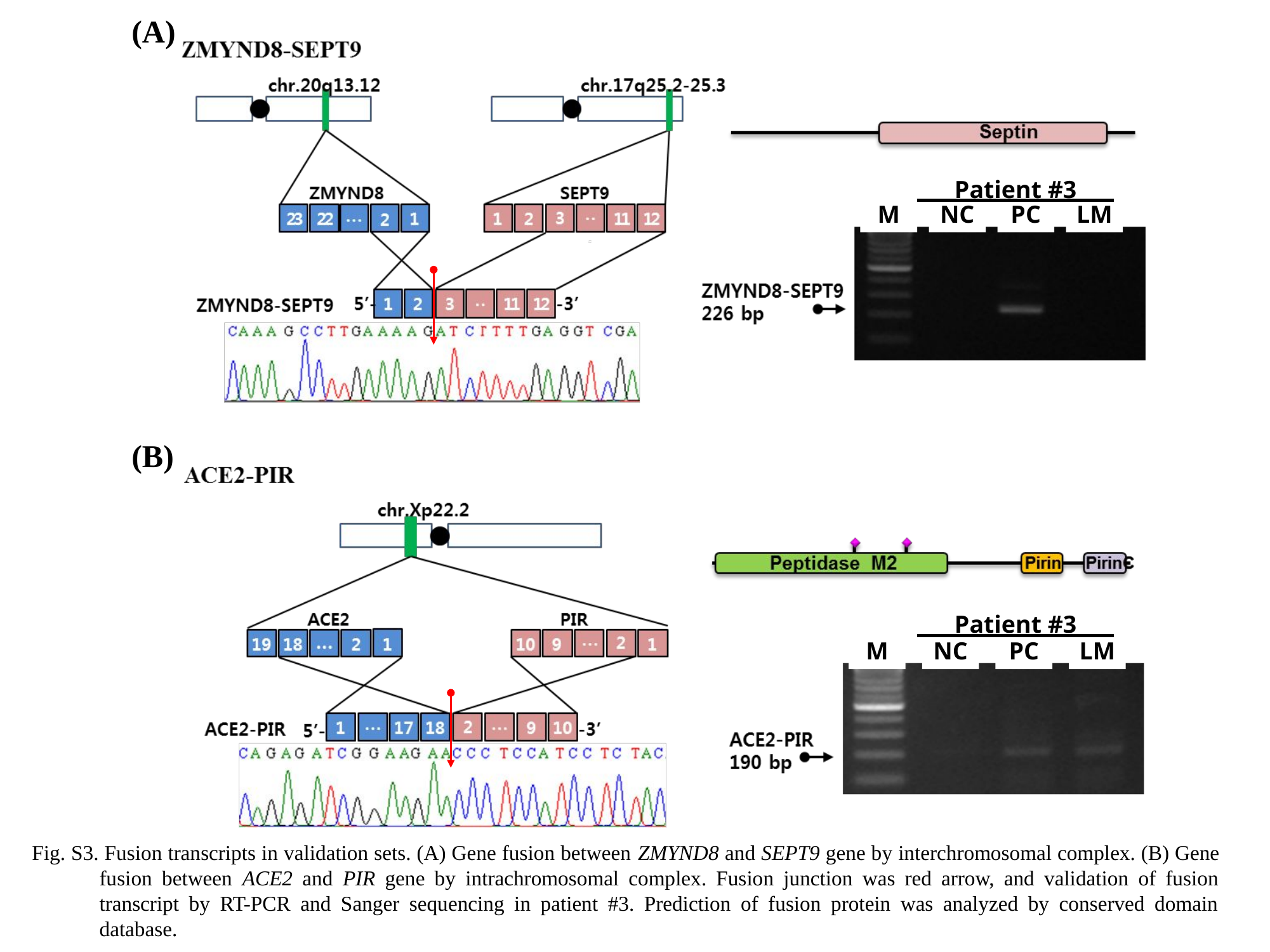

(A)
Patient #3
M
NC
PC
LM
(B)
Patient #3
M
NC
PC
LM
Fig. S3. Fusion transcripts in validation sets. (A) Gene fusion between ZMYND8 and SEPT9 gene by interchromosomal complex. (B) Gene fusion between ACE2 and PIR gene by intrachromosomal complex. Fusion junction was red arrow, and validation of fusion transcript by RT-PCR and Sanger sequencing in patient #3. Prediction of fusion protein was analyzed by conserved domain database.
