# Supplementary material for: Transcriptome analysis of paired primary colorectal carcinoma and liver metastases reveals fusion transcripts and similar gene expression profiles in primary carcinoma and liver metastases
Source: BMC Cancer. 2016 Jul 26;16:539. doi: 10.1186/s12885-016-2596-3 (PMC4962348; doi:10.1186/s12885-016-2596-3)
Supplement: Additional file 8: Figure S4. — RNF43-SUPT4H1 fusion transcript frequency in 10 paired colorectal cancer and normal tissues. M, size marker; N, normal; T, tumor. [file 12885_2016_2596_MOESM8_ESM.pptx]

## Slide 1
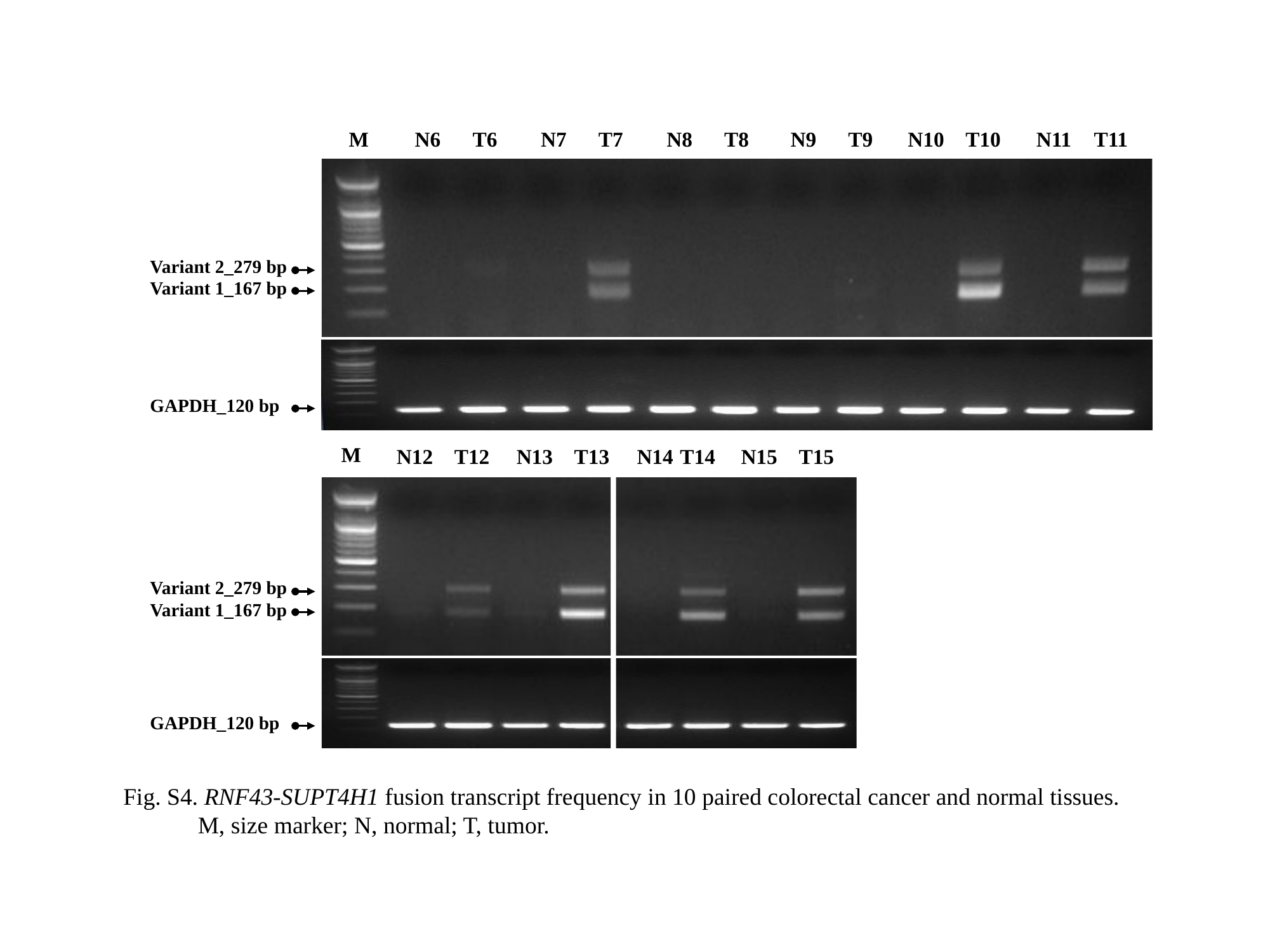

M
N6
T6
N7
T7
N8
T8
N9
T9
N10
T10
N11
T11
M
N12
T12
N13
T13
T14
N15
N14
T15
Variant 2_279 bp
Variant 1_167 bp
GAPDH_120 bp
Variant 2_279 bp
Variant 1_167 bp
GAPDH_120 bp
Fig. S4. RNF43-SUPT4H1 fusion transcript frequency in 10 paired colorectal cancer and normal tissues. M, size marker; N, normal; T, tumor.
