# Supplementary material for: Transcriptome analysis of paired primary colorectal carcinoma and liver metastases reveals fusion transcripts and similar gene expression profiles in primary carcinoma and liver metastases
Source: BMC Cancer. 2016 Jul 26;16:539. doi: 10.1186/s12885-016-2596-3 (PMC4962348; doi:10.1186/s12885-016-2596-3)
Supplement: Additional file 9: Figure S1. — Hierarchical clustering of expression profiles. Data are pre-sented in a matrix format, in which each row represents an indi-vidual gene and each column represents a different tissue sam-ple. Each cell in the matrix represents the expression level of a gene feature in an individual tissue sample. Red, high expres-sion; green, low expression. N, normal colon; C, primary carci-noma; LM, liver metastases; NL, normal liver. (PPTX 1128 kb) [file 12885_2016_2596_MOESM9_ESM.pptx]

## Slide 1
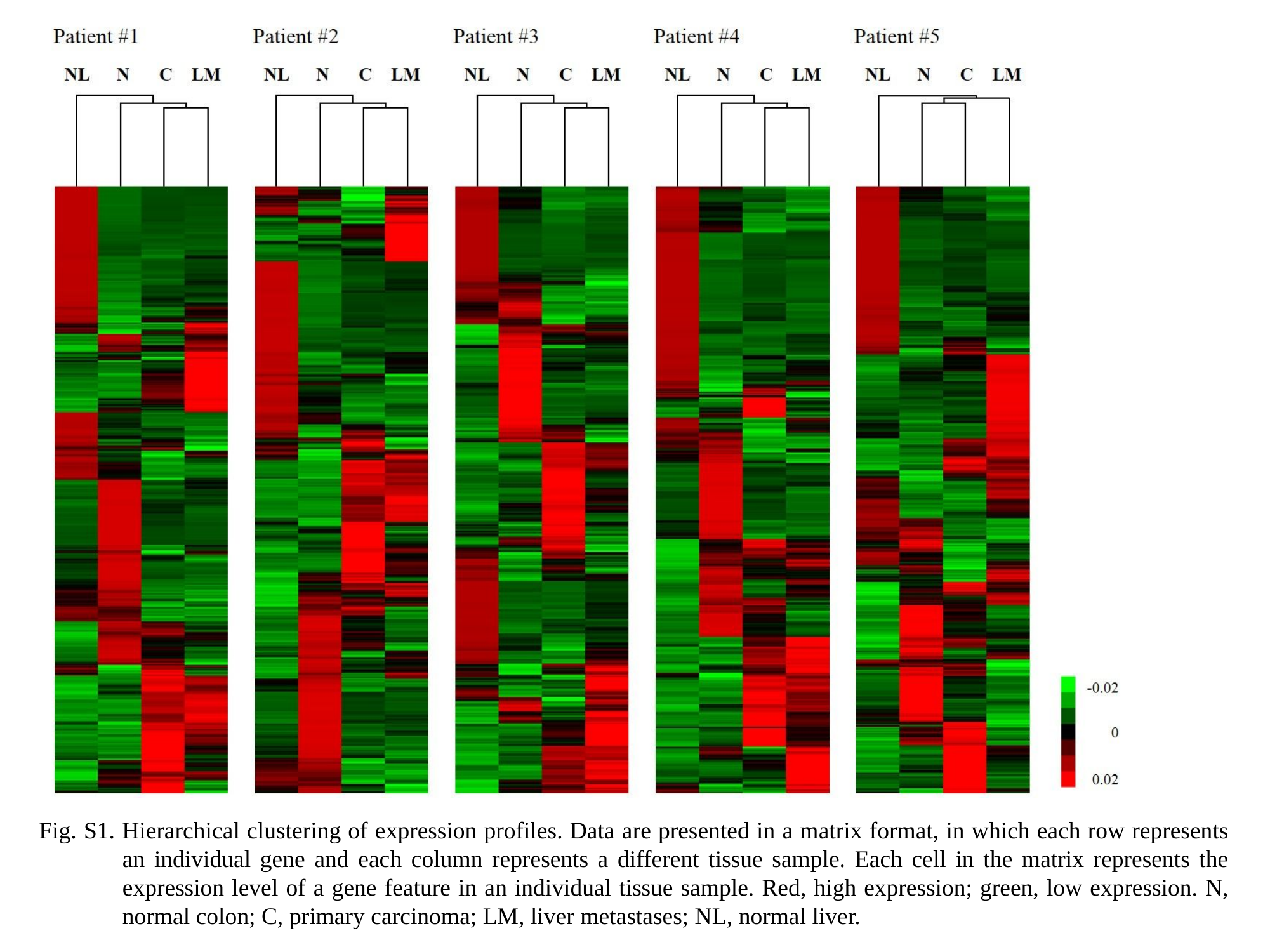

Fig. S1. Hierarchical clustering of expression profiles. Data are presented in a matrix format, in which each row represents an individual gene and each column represents a different tissue sample. Each cell in the matrix represents the expression level of a gene feature in an individual tissue sample. Red, high expression; green, low expression. N, normal colon; C, primary carcinoma; LM, liver metastases; NL, normal liver.
